# The Association of Muscle Dysmorphia, Social Physique Anxiety, and Body Checking Behavior in Male College Students With Weight Exercise

**DOI:** 10.3389/fpsyg.2021.726032

**Published:** 2021-09-24

**Authors:** Yu Zheng, LiFeng Zhang, Ping Shao, XueYing Guo

**Affiliations:** ^1^School of Economics and Management, Chengdu Sport University, Chengdu, China; ^2^Faculty of Basketball and Volleyball, Chengdu Sport University, Chengdu, China; ^3^Sichuan Institute of Sports Science, Sichuan Anti Doping Agency, Chengdu, China; ^4^Department of Student Affairs, Chengdu Sport University, Chengdu, China

**Keywords:** weight exercise, muscle dysmorphia, social physique anxiety, body checking behavior, male college students with weight exercise

## Abstract

**Objectives:** To investigate the association between muscle dysmorphia (MD), social physique anxiety, and body-checking behavior in male college students with weight exercise, and to reveal the association between them.

**Methods:** A total of 492 male college students with weight exercise from 18 Fitness Clubs or bodybuilding centers in Chengdu, China, participated in this study.

**Results:** First, the social physique anxiety scores, body checking frequency, and weight exercise behavior (i.e., frequency, time, and intensity) in male college students with MD were significantly higher than those without MD; it indicated that the higher the exercise frequency they had, the longer the exercise time they cost, and the higher exercise intensity carried out, and the higher the social physique anxiety scores tended to be, the higher the frequency of body checking on “global muscles,” “chest and shoulder muscles,” “comparison with others” and “posture measurement” they did. Second, the mediating effect of the social physique anxiety on MD and body checking was established in the “MD → global muscle checking,” “MD → chest and shoulder muscle checking,” “MD → comparison with others,” and “muscle dysmorphia → posture measurement.”

**Conclusion:** Male college students with MD not only have a higher social physique anxiety, but also a higher frequency of body-checking behavior than the ordinary individuals. Social physique anxiety is one of the important mediating factors to those with MD which affects the body-checking behavior.

## Introduction

Most young male adults always crave big arms, broad shoulders, thick chest muscles, rock-hard abdominal muscles, and hope to develop an inverted triangle-shaped body type (Jin et al., 2015a; Sandgren et al., 2019; Blashill et al., 2020). They are, therefore, commonly dissatisfied with their shortfall of muscles, which can result in negative emotions such as worry, anxiety, upset, or behaviors for altering their muscle shape (Greenway and Price, 2018). To meet their requirements, they often perform high-intensity strength exercises or even taking steroid-assisted drugs to improve muscle. The tendency that focuses on body shape and then causes dissatisfaction and even further results in some anxiety and excessive body-checking behavior, which is called muscle dysmorphia (MD) (Santarnecchi and Dèttore, 2012; Longobardi et al., 2017; Compte et al., 2019). Different from other mental disorders, the MD has its diagnostic criteria: fear of underdeveloped muscles and excessive body-checking behaviors (e.g., the implementation of high-intensity muscle exercise), refusal of important gatherings owing to fear of missing exercise sessions, avoiding exposure to the public, fear of underdeveloped muscles causing depression symptoms. Even if it has been known that muscle exercise will cause injury, it is still enforced implementation by extra muscle increase by improper means, such as taking steroids (Nieuwoudt et al., 2012). Mitchell et al. (2016) also found that those with weight exercise to reduce fat more often take emetic and diuretic medication behavior than those who merely want to lose weight. Carfi et al. (2008) found that males with MD, in addition to more dissatisfaction with muscle mass compared with those without MD, rely heavily on weight exercise to change muscle size and shape. The frequency of mood disorders, eating disorders, or obstructing daily activities was also higher than those without MD. Besides, male college students with MD who monitor the development of muscles, such as muscle size via mirrors, were also higher than those without MD (Sokolova et al., 2013; Tod and Edwards, 2015).

When males with MD feel poorly in some public places and are worried about being ridiculed or criticized, they show a tendency to become anxious or nervous. This psychological reaction is called social physique anxiety, which is a subtype of social anxiety (Driediger et al., 2016; Möllmann et al., 2017). Hildebrandt et al. (2006) found that males with MD had higher scores in body dissatisfaction, body anxiety, muscle building, taking diuretics, and eating high-protein supplements than those without MD. Nieuwoudt et al. (2012) developed an integrated model to characterize the MD behavior, which suggested that males with MD are not only dependent on muscle exercise, but also on the effect of taking drugs to strengthen exercise effect. Besides, it will develop the dieting behavior to reduce the percentage of body fat, then deliberately check his body, such as often weighing, using a mirror to check the appearance, pinching the muscles of a specific site to check the degree of obesity, such behavior is called body checking behavior. That is, multiple methods are used repeatedly to understand, feel, or check physical shape and size (Legenbauer et al., 2017). The purpose of body checking is to allow the individual to obtain shape or size self-perception from an actual physical shape (Mölbert et al., 2017).

In summary, those with MD tend to focus on the degree of muscle build-up, and they will have negative emotions and excessive exercise of muscles due to dissatisfaction, and the core characteristics of most males with MD are excessive attention on body shape, with higher social anxiety symptoms (López et al., 2013; White and Warren, 2014). When an individual wants to change or check his or her body shape, the higher the social physique anxiety the individual has, the higher the frequency of checking the global appearance and the status of specific sites the individual has (Cox et al., 2011; Masuda et al., 2015). MD can reflect certain levels of muscle craving, so that internal desire may induce social physique anxiety, and then high-frequency body checking behavior. However, so far, the relationship between these variables is not very clear. For example, individuals with body dysmorphia often look for mirrors in public places to look at their appearances and stand in front of the mirror for longer than ordinary people (Tod and Edwards, 2015; Bégin et al., 2019). Men who lose weight or have too little muscle due to obesity have a higher score for social physique anxiety and a higher frequency of body checking (Baghurst and Lirgg, 2009). Nowadays, more and more men are involved in weight exercise for changing their body shape. However, research on weight exercise performers with MD is not much (Jin et al., 2015b; Zhu et al., 2018) in China. Unlike weightlifters or bodybuilders, ordinary young adults, especially male college students, perform weight exercises for muscle hypertrophy, not for improving muscle fitness. When the weight exercise performers pay more psychological tendency to muscle mass during exercise, it is prone to MD and higher social physique anxiety problems. However, the underlying mechanism of the muscle size and muscle symmetry checking behavior is still unclear. And compared with other research such as exercise fatigue, fitness, there is little relevant research on whether the weight exercise performers are MD, or there are any differences in the frequency of muscle checking. Besides, whether social physique anxiety has a mediating effect on the causal effects of MD on body-checking behavior has not been reported in the relevant research literature.

As discussed above, we provided the following hypotheses: Hypothesis 1: There are significant correlations in male college students who perform strength training exercise with weights between MD, social physique anxiety, and body-checking behavior. Hypothesis 2: Muscle dysmorphia significantly correlates with body-checking behavior. Hypothesis 3: Social physique anxiety is the mediating effect on muscle dysmorphia and body-checking behavior.

## Methods

### Subjects

(1) The subjects limited to college students from 18 physical fitness centers in Chengdu, China, participated in this study. Survey time: March 2018 to May 2018. During the survey period, investigators randomly selected four days from Monday to Sunday, and randomly choose one of the four periods, i.e., 07:00–08:00 a.m., 9:00–12:00 a.m., 1:00–5:00 p.m., and 6:00–10:00 p.m., to wen to the survey site to investigate.
(2) Before the formal investigation, first, contacted the fitness club managers and obtained the oral consent of the subjects, then arranged the survey time. When surveying, at the entrance of the fitness club, the investigators verbally asked the participants for their permission. After their oral consent, the investigators brought them to the office to fill in the questionnaire separately. Before filling in, the investigator took about 3 min to explain the purpose of the study and the survey instructions, and the participants provided their written informed consent to participate in this study, and then fill out the questionnaire for about 10 min.
(3) After analyzing and processing the questionnaires from the surveys of 520 weight trainees from 18 fitness centers, the data preparation showed that 16 questionnaires were not filled out as required, and 12 were excluded because they were athletes in bodybuilding, weightlifting, or other sports events. Finally, 492 valid questionnaires remained.

This study involving human participants was reviewed and approved by Chengdu Sport University (No. 2020.24).

### Instruments

The research instrument in this study is a self-report questionnaire consisting of four parts:

(1) The demographic information

For this part, the personal information of the subject such as age, height, weight, frequency of participation in strength training, participation time, the intensity of participation in training was listed.

(2) The Muscle Dysmorphia Scale

A 15-item scale developed by Pope et al. (2005) and translated by two English teachers into Chinese was used to measure the intrinsic tendency of excessive anxiety about muscle development and anxiety, for example, whether you often worry that your body is not strong enough and your muscles are not developed enough. Use “Yes” or “No” to evaluate and score. If the subject selected “Yes” for each item, he will get 1 point. If the subject selected “No,” he will get 0 points. According to the diagnostic criteria provided by Pope et al. (2005), if the total score is ≥8 points, the subject is regarded as MD, if <8 points, as non-MD. In terms of reliability, the overall Cronbach's α value was 0.84. In the absolute fit measurement model, *X*^2^ = 2.85, *P* = 0.071 > 0.05, it indicated that the causal path diagram was consistent with the observed data. Besides, adjusted goodness-of-fit index (AGFI), comparative fit index (CFI), normed fit index (NFI), and incremental fit index (IFI) were 0.93, 0.96, 0.94, and 0.95, respectively, and all of them were greater than the criteria of 0.9; the root mean square of error approximation (RMSEA) was 0.041 (< 0.05 for a good fit). In short, the reliability and validity of this scale were acceptable (Table 1).

(3) The Social Physique Anxiety Scale

The Chinese version of the social physique anxiety scale (Hart et al., 1989) revised by Lu and Hung (1999) was used. This scale measures the individual when his/her body shape was observed if there was a tendency to be uneasy or anxious. A total of 12 items (seven items in a positive direction and five items in a reverse direction), for example, “In front of others, I will worry about my body shape.” Using the 5-point Likert scale, the scoring criteria were as follows: “strongly disagree” with a score of 1, “strongly agree” with a score of 5, and a total score between 12 and 60 points. The higher the score one gets, the higher the tendency to be restless or anxious one has. In terms of reliability, the overall Cronbach's α value was 0.87; in the absolute fit value of the measurement model, *X*^2^ = 1.87, *p* = 0.246 > 0.05, it indicates that the path diagram is consistent with observed data. Besides, the fit indices of AGFI, CFI, NFI, and IFI were 0.95, 0.96, 0.96, and 0.95, respectively, and all of them were greater than the criteria of 0.9; the RMSEA was 0.041 (< 0.05 for a good fit). In short, the reliability and validity of this scale were acceptable (Table 1).

(4) The Body Checking Behavior Scale

Using the scale compiled by Hildebrandt et al. (2010) and translated by two English teachers into Chinese, the scale consists of four dimensions with a total of 15 items: the first dimension is the global muscle checking (five items) and the second dimension is the chest and shoulder muscle checking (three items), the third dimension is the comparison with others (three items), and the fourth dimension is the posture measurement (four items). A 5-point Likert scale was adopted: “never” = 1, “rarely” = 2, “sometimes” = 3, “often” = 4, “always” = 5; the total score was between 15 to 75 points, and the higher the score one gets, the higher the frequency of checking the body's behavior one has. After a pre-test factor analysis, four common factors were extracted from this scale, in which the total variance explained was 77.24%, and the overall measurement model test results were: X^2^ = 2.06, *p* = 0.092 > 0.05. It showed that the causal diagram for path analysis was consistent with actual data. Besides, the fit indices of AGFI, CFI, NFI, and IFI were 0.97, 0.95, 0.97, and 0.94, respectively, and all of them were greater than the criteria of 0.9; the RMSEA was 0.025 (< 0.05 for a good fit). The total scale Cronbach's α value was 0.91, the Cronbach's α values of four dimensions were 0.81, 0.87, 0.83, and 0.87, respectively; the values of composite reliability were 0.84, 0.82, 0.81, and 0.86, respectively. In short, the reliability and validity of this scale were acceptable (Table 1).

**Table 1:** The statistics table of quality analysis of three measuring scales.

**Constructs**	**Dimension**	**KMO and Bartlett test**	**Items**	**% of variance**	**Cumulative %**	**CR**	**Cronbach** **α**
Muscle dysmorphia	Uni-dimension	-	15	-	-	0.84	0.84
Validation results of measurement model: *X^2^* = 2.85, *P* = 0.071; AGFI = 0.93, CFI = 0.96, NFI = 0.94, IFI = 0.95, RMSEA = 0.041							
Social physique anxiety	Uni-dimension	-	12	-	-	0.84	0.87
Validation results of measurement model: *X^2^* = 1.87, *p* = 0.246; AGFI = 0.95, CFI = 0.96, NFI = 0.96, IFI = 0.95, RMSEA = 0.041							
Body checking behaviors	global muscle checking	KMO = 0.92*P* < 0.05	5	27.54	27.54	0.84	0.81
	Chest and shoulders checking		3	21.31	48.85	0.82	0.87
	Comparison with others		3	15.41	64.26	0.81	0.83
	Body measurement checking		4	12.98	77.24	0.86	0.87
Validation results of measurement model: *X^2^* = 2.06, *P* = 0.092; AGFI = 0.97, CFI = 0.95, NFI = 0.97, IFI = 0.94, RMSEA = 0.025							

*CR, composite reliability*.

### Statistical Analyses

The IBM SPSS Statistics 21.0 and AMOS 22.0 statistical analysis software package were used. Descriptive analysis, contingency analysis (chi-square test), Pearson product-moment correlation analysis, mediating effect analyses and one-way ANOVA have been carried out to test relevant indicators such as MD and body checking behavior. The test of normality by the skewness and kurtosis values of the continuous variables had been performed, The composite reliability (CR) and internal consistency reliability have been carried out for each scale. The three steps regression methods were used to further reveal the mediating effect of social physique anxiety between MD and body checking. The Sobel's *t*-test (Sobel, 1982) for a significance level of medication effect was carried out. The significance level of all indicator variables in this study was set to α = 0.05.

## Results

### The Difference in Weight Exercise Behavior in Male College Students With Muscle Dysmorphia

According to the descriptive statistics, there were 87 male college students with MD in 492 participants [age: mean (M) = 23.2 years, standard deviation (SD) = 2.45; body height: *M* = 172.4 cm, *SD* = 5.7; body mass: *M* = 69.4 kg, *SD* = 6.45]. The proportion of MD in weight exercise performers was 17.7%, Table 2 shows the association analysis of weight exercise behaviors for both groups with or without MD (87 vs. 405) according to the criteria whether the MD total score <8. It has been found that among the 492 weight exercise performers, the highest weekly participation frequency category was “3–5 day/week” (45.1%), and the highest participation time each session category was “31–60 min each session” (52.2%), the highest intensity of each session category was “slightly sweating each session”(38.2%). There was a significant difference in the weight exercise frequency, time, and intensity between with and without MD, and the statistical results of male college students with MD indicated that the exercise frequency was “3–5 day/week,” exercise time: “>60 min/time” and the exercise intensity: “sweating very much.” The ratio of all three aspects in male college students with MD was significantly higher than those without MD.

**Table 2:** Contingency table of weight exercise behaviors for both groups with and without muscle dysmorphia (n [%]).

	**Frequency (d/wk)**			**Times (min/time)**			**Intensity (sweat)**		
	**≤2**	**3–5**	**≥6**	**≤30**	**31–60**	**>60**	**Slightly**	**Much**	**Extremely**
MD	6 (6.9)	57 (65.5)	24 (27.6)	4 (4.6)	34 (39.1)	49 (56.3)	5 (5.7)	41 (47.1)	41 (47.1)
Non-MD	209 (51.6)	165 (40.7)	31 (7.7)	144 (35.6)	223 (55.1)	38 (9.4)	183 (45.2)	141 (34.8)	81 (20.0)
Total	215 (43.7)	222 (45.1)	55 (11.2)	148 (30.1)	257 (52.2)	87 (17.7)	188 (38.2)	182 (37.0)	122 (24.8)

*MD, muscle dysmorphia; Difference test in exercise frequency for groups with and without muscle dysmorphia: Pearson X^2^ = 67.95, p < 0.001; Difference test in exercise time for groups with or without muscle dysmorphia: Pearson X^2^ = 115.55, P < 0.001; Difference test in exercise intensity for groups with and without muscle dysmorphia: Pearson X^2^ = 201 53.34, p < 0.001; d/wk: day/week, min: minute*.

### Differences in Body Checking Behaviors With and Without Muscle Dysmorphia

Table 3 shows the analysis of differences in body checking and physique anxiety scores for both groups with and without MD. It has been found that there was a significant difference in the frequency of body checking between male college students with and without MD (Pearson *X*^2^ = 38.23, *P* < 0.05), which showed that the proportion of “often checking” of male college students with MD was significantly higher than those without MD (41.4 vs. 15.8%); there were also significant differences in muscle checking sites for male college students with and without MD, and it showed that all four aspects of “global muscle checking,” “chest and shoulder muscle checking,” “comparison with others” and “body measurement test” scores in male college students with MD were significantly higher than those without MD.

**Table 3:** Contingency table on body checking and the descriptive statistics of physique anxiety scores between and without muscle dysmorphia.

	**Frequency of body checking (n [%])**			**Muscle checking sites (points)**			
	**Often**	**Sometimes**	**Occasionally**	**Global**	**Chest and shoulder**	**Comparison with others**	**Posture measurement**
MD	36 (41.4)	27 (31.0)	24 (27.6)	19.61 ± 3.40**	9.92 ± 2.13**	9.81 ± 2.43**	13.56 ± 2.74**
Non-MD	64 (15.8)	98 (24.2)	243 (60.0)	15.15 ± 3.41	7.33± 2.40	6.94 ± 2.26	10.54 ± 2.78
Total	100 (20.3)	125 (25.4)	267 (54.3)	15.94 ± 3.81	7.79 ± 2.55	7.45 ± 2.53	11.07 ± 3.00

*MD, muscle dysmorphia; Independent sample t-test, compared to male college students without muscle dysmorphia:*

** *p < 0.01*.

### The Differences in Social Physique Anxiety and Muscle Checking Sites in Different Exercise Behaviors

Table 4 shows the differences in social physique anxiety and muscle checking sites for different weight exercise behaviors. It has been found that there were significant differences in social physique anxiety among different weight exercise behaviors (F values were 8.69, 7.26, and 6.27, respectively, all *ps* < 0.05). This result showed that first, the higher the social physique anxiety, the higher the weekly frequency of exercise; second, the longer each exercise session had, the higher the intensity of each exercise session; third, the “global muscle checking” behavior of different weight exercise behaviors (F values were 4.89, 6.23, and 5.36, respectively, all *ps* < 0.05), “chest and shoulder muscle checking” behavior (F values were 3.86, 4.02, and 5.79, all *Ps* < 0.05) and “measurement of body posture” (F values were 4.81, 3.87, and 5.08, respectively, all *ps* < 0.05) were significant differences. The result also showed that the higher the scores of “global muscle checking,” “chest and shoulder muscle checking” and “measurement of body checking” behavior, the higher the exercise frequency, the longer the exercise time, and the higher the exercise intensity.

**Table 4:** Differences in social physique anxiety scores and muscle checking sites for different weight exercise behaviors (Mean ± Standard Deviation).

**Variables**	**Category**	**Social physique anxiety**	**global muscle checking**	**Chest and shoulder**	**Comparison with others**	**Posture measurement**
Frequency (d/wk)	≤ 2 (a)	36.07 ± 8.66	15.32 ± 5.08	7.52 ± 2.89	7.89 ± 2.47	10.15 ± 4.03
	3–5 (b)	42.96 ± 11.32	17.35 ± 6.38	8.01 ± 2.80	8.48 ± 2.49	11.19 ± 3.74
	≥ 6 (c)	48.94 ± 10.58	19.47 ± 5.78	10.36 ± 3.03	8.77 ± 2.26	14.81 ± 4.79
	F-value:MC	8.69*:c>b>a	4.89*:c>b>a	3.86*: c>b=a	0.15: c=b=a	4.81*: c>b=a
Time (min/time)	≤ 30 (a)	37.98 ± 11.21	16.10 ± 4.98	7.39 ± 3.36	7.95 ± 4.77	10.49 ± 4.64
	31–60 (a)	40.51 ± 10.05	16.88 ± 5.56	7.88 ± 3.01	8.34 ± 2.98	11.11 ± 3.75
	> 60 (a)	47.66 ± 9.66	19.16 ± 7.31	9.87 ± 3.66	8.85 ± 3.23	14.55 ± 5.71
	F-value: LSD	7.26*:c>b=a	6.23*: c>b=a	4.02*: c>b=a	0.56: c=b=a	3.87*: c>b=a
Intensity (sweat)	Slightly (a)	37.73 ± 6.25	15.12 ± 5.02	6.60 ± 4.15	7.77 ± 3.61	10.26 ± 3.11
	Much	38.57 ± 8.89	16.77 ± 6.27	8.27 ± 3.77	8.36 ± 2.37	10.87 ± 3.64
	Extremely	49.85 ± 10.24	20.25 ± 5.21	10.21 ± 4.68	9.01 ± 2.78	15.02 ± 4.63
	F-value: Scheffe test	6.27*: c>b=a	5.36*: c>b=a	5.79*: c>b>a	1.58: c=b=a	5.08*: c>b=a

*MC: multiple comparisons, b > a indicates group b is significantly higher than group a, and b = a indicates groups a and b are not statistically significant difference;*

* *p < 0.05*.

### The Association Between Social Physique Anxiety, Muscle Dysmorphia, and Body-Checking Behavior

Table 5 shows the correlation relationship between MD, social physique anxiety, and body checking behaviors. It has been found there was a significant positive correlation between MD and social physique anxiety, four dimensions of body checking (*r* = 0.47, 0.59, 0.51, 0.55, and 0.61, respectively, all *ps* < 0.05); there was a significant positive correlation between social physique anxiety and body checking behaviors of four dimensions (*r* = 0.51, 0.45, 0.41, and 0.40, respectively, all *ps* < 0.05); body checking behaviors of four dimensions were highly correlated with each other and were not independent. The above results indicated that social physique anxiety was possible as a mediating effect on MD and body checking behavior.

**Table 5:** Descriptive statistics and Pearson product-moment correlation across muscle dysmorphia, social physique anxiety, and body checking behaviors (*N* = 492).

**Dimensions**	**MD**	**SPA**	**GME**	**CSC**	**CWO**	**PME**	**Mean ± Std. deviation**
MD	1.00						5.15 ± 2.69
SPA	0.47*	1.00					42.64 ± 7.66
GME	0.59**	0.50*	1.00				17.38 ± 4.77
CSC	0.51*	0.45*	0.68**	1.00			8.63 ± 2.65
CWO	0.55*	0.41*	0.62**	0.79**	1.00		8.38 ± 2.74
PME	0.61**	0.40*	0.71**	0.66**	0.60**	1.00	12.05 ± 3.55

*MD, muscle dysmorphia; SPA, social physique anxiety; GME, global muscle checking; CSC, chest and shoulder muscle checking; CWO, comparison with others; PME, posture measurement checking;*

* *p < 0.05*,

** *p < 0.01*.

### The Mediating Role of Social Physique Anxiety Between Muscle Dysmorphia and Body Checking Behavior

**Figure 1:**
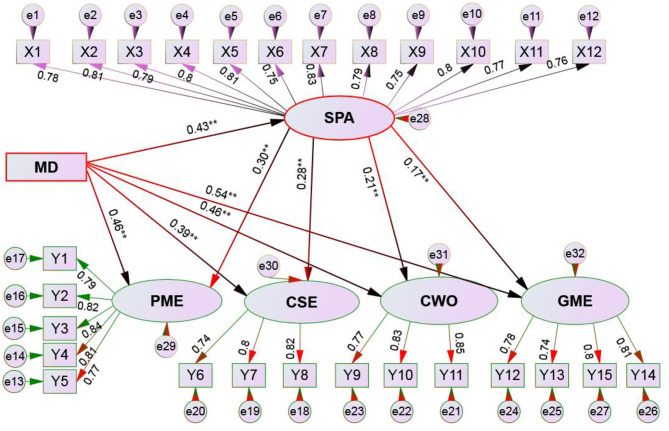
Structural Equation model schematic of muscle dysmorphia, social physique anxiety, and body checking behaviors. MD, muscle dysmorphia; SPA, social physique anxiety; GME, global muscle checking; CSE, chest and shoulder muscle checking; CWO, comparison with others; PME, posture measurement checking. **p < 0.01.

Figure 1 shows that in the baseline model, X^2^/df = 14.01, *P* = 0.081 > 0.05, the overall fit, the goodness of fitness index GFI (0.941), and the adjusted goodness fitness index AGFI (0.927) was >0.90, RMSEA (0.026) was lower than 0.1, and the normative fit index NFI (0.915) and the comparative fit index CFI (0.915) were both >0.90. Based on the above judgments, it showed that the overall fit of the structural model of this study was acceptable (see Figure 1).

According to Zheng and Cai's (2016) method (i.e., bootstrap algorithm), the mediating role of social physique anxiety was explored. Table 6; Figure 1 show:

(1) The mediating model I: MD → social physique anxiety → global muscle checking. The mediating effect was 0.43 × 0.30 = 0.146 (*p* < 0.01), 95% confidence interval (CI) was [0.117, 0.201], which did not cross 0, thus reached a significant level. The performance “social physique anxiety” had a mediating effect between “MD” and “global muscle checking”; the direct effect of MD → the global muscle checking was 0.46 × 0.46 = 0.213, 95% CI [0.200, 0.226], which did not cross 0, thus reached the significant level; according to the bootstrap hypothesis, social physique anxiety was partially mediating role between MD and global muscle checking.
(2) The mediating model II: MD → social physique anxiety → chest and shoulder muscle checking. The mediating effect was 0.43 × 0.28 = 0.120 (*p* < 0.05), 95% CI was [0.095, 0.145], which did not cross 0, thus reached the significant level, indicating social physique anxiety had a mediating effect between “MD” and “chest and shoulder muscle checking”; The direct effect of MD → the chest and shoulder muscle checking was 0.39 × 0.39 = 0.152, and 95% CI was [0.124, 0.180], which did not cross 0, reached a significant level. According to the bootstrap hypothesis, it was inferred that social physique anxiety was a partially mediating role between “MD” and “chest and shoulder muscle checking.”
(3) The mediating model III: MD → social physique anxiety → comparison with others. The mediating effect was 0.43 × 0.21 = 0.090 (*p* < 0.05), 95% CI was [0.006, 0.120], which did not cross 0, thus reached the significant level, indicating that social physique anxiety had a mediating effect between “MD” and “comparison with others”; The direct effect of MD → the comparison with others was 0.46 × 0.46 = 0.212, and 95% CI was [0.187, 0.237], which did not cross 0, thus reached the significant level; according to the bootstrap hypothesis, it was inferred that social physique anxiety was partially mediating role between “MD” and “comparison with others.”
(4) Mediating model IV: MD → social physique anxiety → posture measurement checking. The mediating effect was 0.43 × 0.17 = 0.073 (*p* < 0.05), 95% CI was [0.054, 0.092], which did not cross 0, thus reached a significant level, indicating that social physique anxiety had a mediating effect between “MD” and “posture measurement checking.” The direct effect of MD → posture measurement checking was 0.54 × 0.54 = 0.292 (*p* < 0.05), 95% CI was [0.215, 0.369], which did not cross 0, thus reached a significant level. According to the bootstrap hypothesis, social physique anxiety was a partially mediating role between “MD” and “body measurement test.”

**Table 6:** Mediating effect of social physique anxiety scores on muscle dysmorphia and body checking behaviors.

**ModelI:MD (a) → SPA (b) → GME (c)**			**ModelII:MD (a) → SPA (b) → CSC(d)**			**Model III:MD (a) → SPA(b) → CWO (c)**			**Model IV: MD (a) → SPA(b) → PME (f)**		
**Path**	**Weight**	**95% CI**	**Path**	**Weight**	**95% CI**	**Path**	**Weight**	**95% CI**	**Path**	**Weight**	**95% CI**
ME			ME			ME			ME		
a → b	0.129*	0.112–0.146	a → b	0.12*	0.104–0.187	a → b	0.090*	0.078–0.148	a → bf	0.073*	0.057–0.119
DE			DE			DE			DE		
a → b	0.43*	0.416–0.522	a → b	0.43*	0.416–0.522	a → b	0.43*	0.416–0.522	a → b	0.43*	0.416–0.522
b → c	0.30*	0.175–0.331	b → d	0.28*	0.158–0.314	b → e	0.21*	0.130–0.262	b → f	0.17*	0.095–0.228
a → c	0.46*	0.378–0.510	a → d	0.39*	0.267–0.415	a → e	0.46*	0.334–0.425	a → f	0.54*	0.361–0.454
TE			TE			TE			TE		
a → c	0.341*	0.326–0.356	a → d	0.273*	0.259–0.286	a → e	0.302*	0.202–0.402	a → f	0.364*	0.225–0.504

*MD, muscle dysmorphia; SPA, social physique anxiety; GME, global muscle checking; CSC, chest and shoulder muscle checking; CWO, comparison with others; PME, posture measurement checking; ME, mediating effect; DE, direct effect; TE, total effect; CI, confidence interval;*

* *p < 0.05*.

## Discussion

### The Incidence of Muscle Dysmorphia and Weight Exercise Behavior

The result of this study found that the proportion of MD in weight trainers was similar to the result (17.7 vs. 16.9%) of Hildebrandt et al. (2006). From the exploration of MD, we found that the higher scores in the latent variables were seeking muscle drive, lower tolerance of appearance, and dysfunction, while the higher scores in manifest variables were the desire for muscle hypertrophy, obvious use of muscle supplements behavior. Exercise performance characteristics occurred the three main characteristics (i.e., the higher frequency, the longer time, and the higher intensity). Based on this, it was inferred that weight exercise for developed muscles might have adverse psychological reactions, and therefore must be highly concerned by the fitness center coaches, fitness instructors, or the bodybuilder himself. This study found that the body checking behavior in male college students with MD was significantly higher than that of non-MD (41.4 vs. 15.8%). This supported the conclusion that male college students with MD often worry about underdeveloped muscles (Bégin et al., 2019); that is, when body checking was strengthened by certain behaviors, the male with MD always pay attention to the situation of muscle changes, such as showing the body in front of the mirror and checking the symmetry of the muscles. From the examined muscle sites, male college students with MD in this study scored significantly higher in the “global muscles,” “chest and shoulder muscles,” “comparison with others,” and “posture measurement” than those without MD, and it showed the higher the MD had, the higher the exercise frequency, time and intensity of the global muscle, chest and shoulder muscles tended to be. This result was similar to the study by Greenway and Price (2018). That is, males with MD always considered themselves too emaciated. To monitor the development of muscles, it was prone to indicating improper management behavior, such as often weighing or grasping the tissues of specific sites. Thus, they use a variety of methods to confirm the state of tissues.

Similarly, Babusa et al. (2015) found that the development of muscles was the primary goal of male participation in weight exercise, especially the degree of the event that can show masculinity. For individuals craving muscle hypertrophy in weight exercise, the following was also the incidence in this population: they will take more time to perform the weight exercise, even do it several times a day. Moreover, they were often sweating during exercise. What they have done just met the internal needs of muscle hypertrophy, thus it is easy to lead to the checking behaviors of global muscles, chest and shoulder muscles, and posture measurements.

### The Social Physique Anxiety and Weight Exercise Behavior in Male College Students With Muscle Dysmorphia

This study found that male college students with MD were significantly higher in social physique anxiety scores than those without MD; there was a significant positive correlation between MD and social physique anxiety, which was characterized by the higher social physique anxiety, the higher frequency of weight exercise, the longer exercise time and the higher exercise intensity. The results in the present study were similar to the findings of previous studies (Murray et al., 2012; Lanfranchi et al., 2015; Mitchell et al., 2016). For example, Lanfranchi et al. (2015) found that there was a significant positive correlation between muscle craving and social physique anxiety; Mitchell et al. (2016) also found that the proportion of the dislike of his body shape in ordinary males with weight exercise was high, and also quite long for changing body shape through exercise. Then, it can be speculated that males with weight exercise not only have the pursuit of muscle development but also have higher physique anxiety.

Moreover, Murray et al. (2012) found that to achieve his body shape goal quickly, the male had somewhat subjective consciousness in weight exercise, and often had a higher exercise frequency and a longer exercise duration. It can be seen that social physique anxiety can lead individuals to pay too much attention to the appearance of the body. To avoid experiencing anxiety, some participants exercise on fixed time or exercise alone. Thus, social physique anxiety is likely to be a latent factor in inducing undesirable body shape control behavior.

### The Mediating Role of Social Physique Anxiety on the Relationship Between Muscle Dysmorphia and Body Checking Behavior

From the association between MD, social physique anxiety, and body checking action, MD was highly correlated with social physique anxiety. The four dimensions of body-checking behavior were also highly correlated with MD but moderately related to social physique anxiety. The relationship was similar to previous studies (Walker et al., 2009; López et al., 2013; Longobardi et al., 2017). As López et al. (2013) pointed out that both men and women focus on the global appearance or the status of specific parts, the difference was that men focus on the development of muscle size and shape. Hildebrandt et al. (2006) found that for the male, the higher craving for the degree of muscle build-up, the higher the social physique anxiety will be. Longobardi et al. (2017) pointed out that MD induced higher body type attention, to confirm the global or specific part of the muscle status, the subjects will appear the behaviors of looking in the mirror, weighing or calculating the net weight, and these behaviors were developed to understand or feel the body shape. A study by Walker et al. (2009) confirmed that the body checking was quite private, often appearing in the body-type attention group, and then develop body-shaped control behaviors such as exercising muscles or eating high-protein substances.

This study found that social physique anxiety was established as a mediator of muscle dysmorphia and body checking behavior. Among them, social physique anxiety was established as the mediating effect of “MD → global muscle checking,” “MD → chest and shoulder muscle checking,” “MD → comparison with others,” and “muscle dysmorphia → posture measurement.” In other words, in addition to the various body checking (e.g., global muscles, chest and shoulder muscles checking) in males with MD, which also indirectly affected the subject's body checking behavior through the mediating effect of social physique anxiety, which was similar to the results of Fitzsimmons-Craft et al. (2014). They found that the occurrence of body-checking behavior was related to physique anxiety. To avoid experiencing physique anxiety in the social field, individuals often deliberately examine specific parts of the body, hoping to construct a positive self-image. The results of this study confirmed that physique anxiety affects the global muscle and specific part of the muscle checking behavior, and even encourages participants to “comparison with others” muscle size and conduct “body measurement” behavior (measuring muscle volume), which have not previously proved. Because previous scholars only suggested that males with MD will have an anxiety reaction due to focused muscles being underdeveloped. The more the males with MD pay too much attention to their body shape, the more likely the males with MD are to monitor muscle development, but the males with MD do not suggest that physique anxiety may play an intermediary role (Hildebrandt et al., 2010; Tod and Edwards, 2015; Mitchell et al., 2016).

Finally, in terms of the predictive effect of MD, it was clear that its influence on the four dimensions of body-checking behavior was significantly stronger than that of social physique anxiety. According to Longobardi et al. (2017), males with MD maintained muscle strength and exercise muscles and examined the size or symmetry of specific parts. These were actions derived from excessive attention to body types and muscles, and the typical behavioral characteristics of physical development monitored by males with MD. Since social physique anxiety only reflects the degree of personal anxiety, it is not the actual behavior of the body type control, so it can be inferred that its predictive power for body checking behavior will be lower than MD. The coefficient of determination R^2^ indicated that the MD had the highest interpretation variation (20.25%) for the global muscle checking, which meant that the higher the degree of muscle underdevelopment, the higher the frequency of global muscle checking behavior. The results of the study are similar to Hunt et al. (2013). But Hunt et al. did not find out what special screening behaviors would be induced by MD. However, the global muscle checking behavior in this study has been supported. That is, apart from paying attention to the muscles of the chest, abdomen, and biceps, male college students with MD also focus on the hardness, symmetry, obvious shape of the muscles, and so on.

## Conclusion

In the present study, the data substantiated the three hypotheses that we proposed. Specifically, we found that male college students craving muscle hypertrophy in weight exercise was highly correlated with MD. The social physique anxiety, body checking, and weight exercise behavior (i.e., frequency, time, and intensity) in male college students with MD were significantly higher than those without MD. The higher frequency of participation in weight exercise, longer exercise time, and higher intensity of exercise highly associated with higher MD and social physique anxiety. Finally, in this study, social physique anxiety was established as a mediating effect of MD and body checking behavior in the “MD → global muscle checking,” “MD → chest and shoulder muscle checking,” “MD → comparison with others,” and “muscle dysmorphia → posture measurement.”

### Limitations

Owing to various subjective and objective reasons, this study has some limitations. Above all, after the Chinese translation, the MD questionnaire was pre-tested before the formal investigation, and its validity and reliability were verified. However, it was first used in the fitness clubs in western China, and its applicability needs further practice tests. Second, the body checking behavior questionnaire consists of four dimensions, but the content of the evaluation items overlaps. The subject is prone to confusion during the process of answering. Future research needs to modify the language expression in the questionnaire further and test the scale, and the validity and test-retest reliability should be confirmed. Third, to date, the research on MD in weight exercise is relatively scarce in China. Future research can be based on the behavioral characteristics of male college students participating in weight exercise, and try to develop scales and assessment contents that are in line with China's national conditions, to improve the application. Fourth, Individuals involved in MD are not limited to male weight exercise performers. Thus, future research should select populations from other sports activities, such as triathlon, marathon, and explore the generalized characteristics of MD. Finally, there are other constructs of MD, such as the pursuit of muscle size drive, low appearance tolerance, and dysfunction. Future research can analyze its relationship with social anxiety and body-checking behavior.

## Data Availability Statement

The raw data supporting the conclusions of this article will be made available by the authors, without undue reservation.

## Ethics Statement

Ethical review and approval was not required for the study on human participants in accordance with the local legislation and institutional requirements. The patients/participants provided their written informed consent to participate in this study.

## Author Contributions

YZ wrote this manuscript. LZ, PS, and XG carried the investigation. All authors designed this study and analyzed the data.

## Conflict of Interest

The authors declare that the research was conducted in the absence of any commercial or financial relationships that could be construed as a potential conflict of interest.

## Publisher's Note

All claims expressed in this article are solely those of the authors and do not necessarily represent those of their affiliated organizations, or those of the publisher, the editors and the reviewers. Any product that may be evaluated in this article, or claim that may be made by its manufacturer, is not guaranteed or endorsed by the publisher.
